# Imaging of Human Insulin Secreting Cells with Gd-DOTA-P88, a Paramagnetic Contrast Agent Targeting the Beta Cell Biomarker FXYD2γa

**DOI:** 10.3390/molecules23092100

**Published:** 2018-08-21

**Authors:** Stéphane Demine, Alexander Balhuizen, Vinciane Debaille, Lieke Joosten, Maïté Fereau, Satya Narayana Murthy Chilla, Isabelle Millard, Raphaël Scharfmann, Dominique Egrise, Serge Goldman, Piero Marchetti, Martin Gotthardt, Sophie Laurent, Carmen Burtea, Decio L. Eizirik

**Affiliations:** 1 ULB-Center for Diabetes Research, Medical Faculty, Université Libre de Bruxelles (ULB), Route de Lennik, Brussels 808-CP618, 1070 Brussels, Belgium; a.balhuizen@gmail.com (A.B.); Isabelle.Millard@ulb.ac.be (I.M.); deizirik@ulb.ac.be (D.L.E.); 2 Laboratoire G-Time, Université Libre de Bruxelles (ULB), Av. F.D. Roosevelt 50 CP 160/02, 1050 Brussels, Belgium; vdebaill@ulb.ac.be; 3 Department of Radiology and Nuclear Medicine, Radboud University Medical Center, 6500 HB Nijmegen, The Netherlands; Lieke.Claessens-Joosten@radboudumc.nl (L.J.); martin.gotthardt@radboudumc.nl (M.G.); 4 Department of General, Organic and Biomedical Chemistry, NMR and Molecular Imaging Laboratory, University of Mons, Avenue Maistriau 19, Mendeleev Building, B-7000 Mons, Belgium; fereau.maite@gmail.com (M.F.); satyamurthy.chilla@gmail.com (S.N.M.C.); Sophie.LAURENT@umons.ac.be (S.L.); Carmen.BURTEA@umons.ac.be (C.B.); 5 INSERM U1016, Université Paris-Descartes, Institut Cochin, 75014 Paris, France; raphael.scharfmann@inserm.fr; 6 Center for Microscopy and Molecular Imaging (CMMI), Université Libre de Bruxelles (ULB) and University of Mons, 12 Rue des Professeurs Jeener et Brachet, 6041 Charleroi-Gosselies, Belgium; Dominique.Egrise@erasme.ulb.ac.be (D.E.); sgoldman@ulb.ac.be (S.G.); 7 Department of Clinical and Experimental Medicine, and University Hospital, University of Pisa, 56100 Pisa, Italy; piero.marchetti@med.unipi.it

**Keywords:** peptide-based imaging, beta cell imaging, paramagnetic contrast agent, non-invasive imaging, MRI, pancreatic beta cell, Type 1 diabetes, Type 2 diabetes

## Abstract

Non-invasive imaging and quantification of human beta cell mass remains a major challenge. We performed pre-clinical in vivo validation of a peptide previously discovered by our group, namely, P88 that targets a beta cell specific biomarker, FXYD2γa. We conjugated P88 with DOTA and then complexed it with GdCl_3_ to obtain the MRI (magnetic resonance imaging) contrast agent (CA) Gd-DOTA-P88. A scrambled peptide was used as a negative control CA, namely Gd-DOTA-Scramble. The CAs were injected in immunodeficient mice implanted with EndoC-βH1 cells, a human beta cell line that expresses FXYD2γa similarly to primary human beta cells. The xenograft-bearing mice were analyzed by MRI. At the end, the mice were euthanized and the CA biodistribution was evaluated on the excised tissues by measuring the Gd concentration with inductively coupled plasma mass spectrometry (ICP-MS). The MRI and biodistribution studies indicated that Gd-DOTA-P88 accumulates in EndoC-βH1 xenografts above the level observed in the background tissue, and that its uptake is significantly higher than that observed for Gd-DOTA-Scramble. In addition, the Gd-DOTA-P88 showed good xenograft-to-muscle and xenograft-to-liver uptake ratios, two potential sites of human islets transplantation. The CA shows good potential for future use to non-invasively image implanted human beta cells.

## 1. Introduction

Type 1 diabetes (T1D) is an autoimmune disease leading to the progressive loss of insulin-producing pancreatic beta cells [1,2]. The incidence of T1D is increasing at a fast rate, and it is predicted that new cases of the disease will double in coming decades [3]. The pathogenesis of T1D and the rate of beta cell mass (BCM) loss in the pre- and early-diabetes period remain unknown, hampering attempts to prevent or cure the disease [4]. T1D patients have to either rely on regular insulin injections or, in special cases, on human islet transplantation. To date, these grafts can only be followed for a short-time post-transplantation (by using pre-labelling with radioisotopes [5] (or superparamagnetic particles) and/or functionally evaluated by measuring C-peptide secretion [6], usually several weeks after transplantation in order to allow sufficient vascularization. However, the correlation between C-peptide values and number of transplanted islets is usually poor and depends on several factors (purity of the islet preparation, functionality of the grafted beta cells, etc.). Although promising data have been obtained with other tracers such as exenatide, there are currently no non-invasive methods that allow accurate quantification of the BCM/graft fluctuations over time [7]. Indeed, the specificity and/or sensitivity of previously developed BCM biomarkers and tracers to image BCM have not yet reached sufficient quality to allow their routine use in the stratification and follow up of diabetic patients [8,9,10,11].

In an attempt to develop novel approaches for beta cell imaging, we have used a functional genomics approach that led to the identification of a new beta cell surface biomarker, namely the splice variant FXYD2γa [12]. FXYD2γa expression is restricted to beta cells in both humans and *Macacus cynomolgus* [12], suggesting the possibility to use this biomarker for specific quantification of human BCM. Based on this finding, we generated a peptide (P88) with high specificity for FXYD2γa [13]. When coupled to ultra-small superparamagnetic particles of iron oxide (USPIO, an MRI contrast agent), P88 could be used for in vivo MR imaging of FXYD2γa-expressing cells in a tumor (non-beta cell) model [13]. This initial study, however, was (1) restricted to a non-physiological cell model, unrelated to beta cells [13], and (2) P88 was conjugated to a contrast agent (CA) that produces a negative contrast on MRI. This CA is partially excreted via the reticuloendothelial system (RES) despite a poly (ethylene glycol) (PEG) coating [14,15]. This RES uptake, in combination with the well-known macrophage infiltration of pancreatic islets, may cause an inaccurate BCM reading [15].

Here, we conjugated P88 to a paramagnetic MRI CA comprising a chelator (1,4,7,10-tetraazacyclododecane-1,4,7,10-tetraacetic acid, DOTA) complexed with GdCl_3_ to solve eventual limitations of the superparamagnetic CAs and pave the way towards future clinical implementation. Gadolinium (Gd)-based CAs (GBCA) are routinely employed in radiology as diagnostic agents. Due to their transient extracellular distribution, fast renal excretion and rare adverse reactions, GBCA are considered safe for clinical practice although some reports suggest that patients with impaired renal function may develop nephrogenic systemic fibrosis (NSF) after exposure to GBCA, especially when exposed to larger doses than clinically recommended (i.e., 0.1 mmoL/kg b.w.) [16,17]. Recent pre-clinical studies have shown that long-term GBCA-associated Gd retention is largely unaffected by renal function [17], and that Gd-DOTA does not affect renal function in patients with chronic kidney disease [18].

This novel CA was first validated by imaging mice implanted with a genetically manipulated Chinese hamster ovary (CHO) cell line that overexpresses FXYD2γa. Next, the FXYD2γa-targeted CA was employed to image in vivo human insulin secreting cells implanted into mice. The outcome of this study is the validation of a beta cell specific MRI CA that might be useful for future non-invasive imaging of human insulin-secreting cells, pending further optimization.

## 2. Results

**Figure 1:**
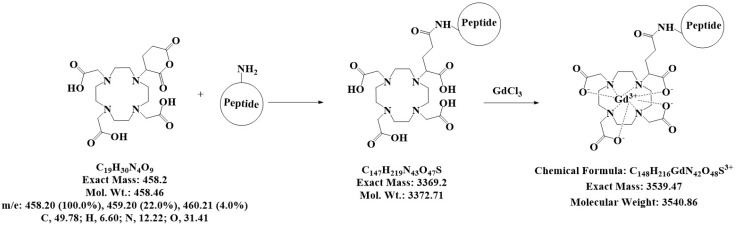
Coupling of the peptides to DOTA. Scheme for the synthesis of the two contrast agents (CA), namely Gd-DOTA-P88 and Gd-DOTA-Scramble, which were produced by coupling P88 or Scramble to DOTA, and thereafter complexing it with GdCl_3_ to prepare the CA for MRI. The characteristics of the chemical structures are outlined beneath the reaction.

**Figure 2:**
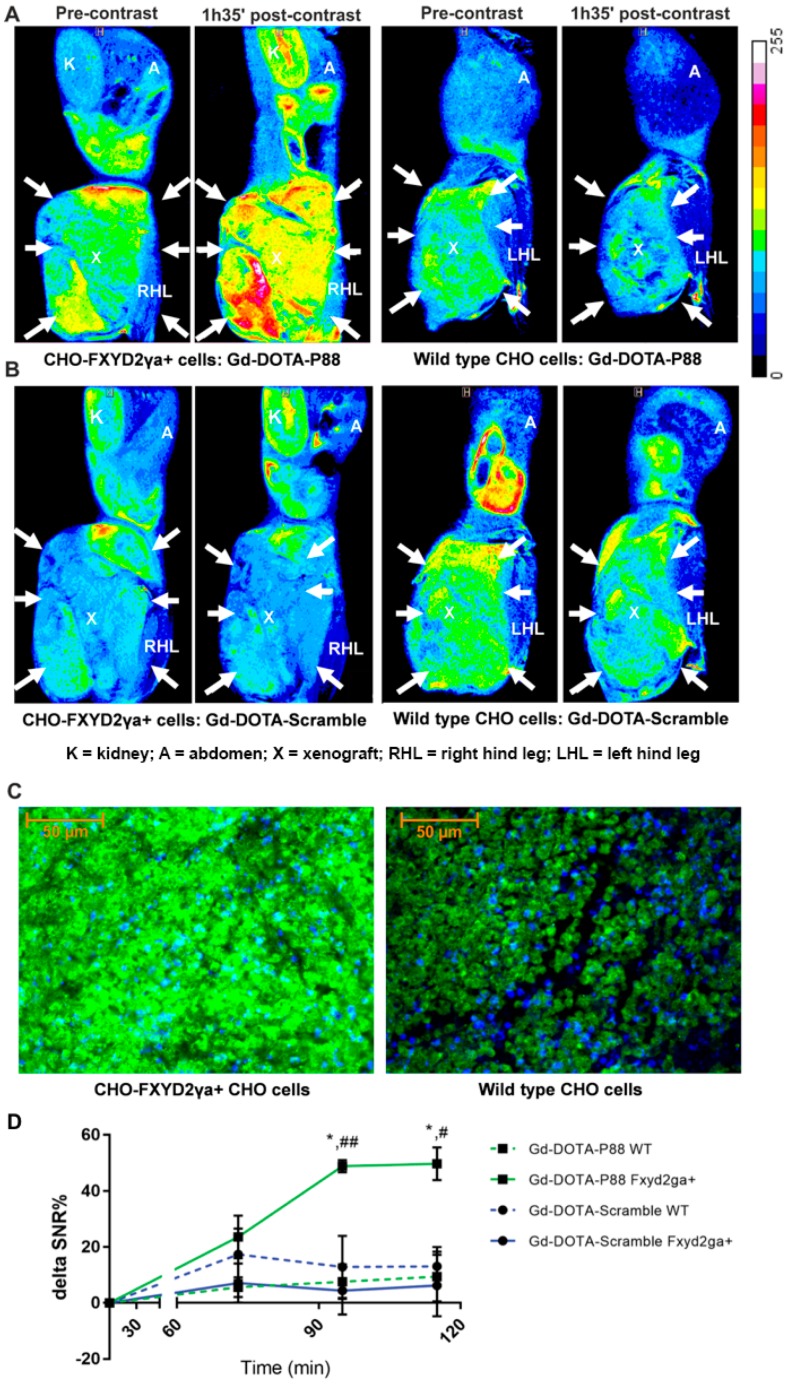
MR imaging of Gd-DOTA-P88 and Gd-DOTA-Scramble in mice implanted with CHO-FXYD2γa^+^ and wildtype CHO cells. (**A**,**B**) Representative color overlay of MR images of mice bearing CHO FXYD2γa^+^ or wildtype CHO xenografts. Pre-contrast images were acquired before the injection of CAs while the post-contrast images were obtained 95 min after i.v. administration of 0.1 mmoL Gd/kg b.w. of Gd-DOTA-P88 (**A**) or Gd-DOTA-Scramble (**B**). Mice were implanted with CHO-FXYD2γa^+^ in the right hind leg and with wildtype CHO cells in the left hind leg (xenografts are indicated by arrows). The images are representative for images obtained in 3–4 mice. (**C**) The FXYD2γa expression in CHO-FXYD2γa^+^ and wildtype CHO xenografts was confirmed by immunofluorescence, where the biomarker is stained in green with fluorescein and the nuclei are stained in blue with DAPI. (**D**) Region of Interest (ROI) quantification of the xenografts on the acquired images. The contrast enhancement is expressed as ΔSNR%, Gd-DOTA-P88 is shown in green and Gd-DOTA-Scramble in blue; a solid line indicates CHO-FXYD2γa^+^ while the dashed line indicates wildtype (wt) CHO cells. The data are expressed as means ± SD, *n* = 3–4 mice in each group; * *p* ≤ 0.05: Gd-DOTA-P88-FXYD2γa^+^ vs. Gd-DOTA-P88-wt; # *p* ≤ 0.05, ## *p* ≤ 0.01: Gd-DOTA-P88-FXYD2γa^+^ vs. Gd-DOTA-Scramble-FXYD2γa^+^; Two-way ANOVA with Šídák correction for multiple tests. Legend: K = kidney; A = abdomen; X = xenograft; RHL = right hind leg; LHL = left hind leg.

After the CA synthesis (Figure 1), the in vivo imaging properties of Gd-DOTA-P88 were first evaluated in a mouse model bearing both wild type CHO cells and CHO-FXYD2γa^+^-cells. The CA showed clear FXYD2γa^+^-specific contrast enhancement over both the control peptide and the wild type cells (Figure 2A,B,D and Figure S1). The FXYD2γa expression was confirmed by immunofluorescence and qPCR (Figure 2C; Figure S2).

**Figure 3:**
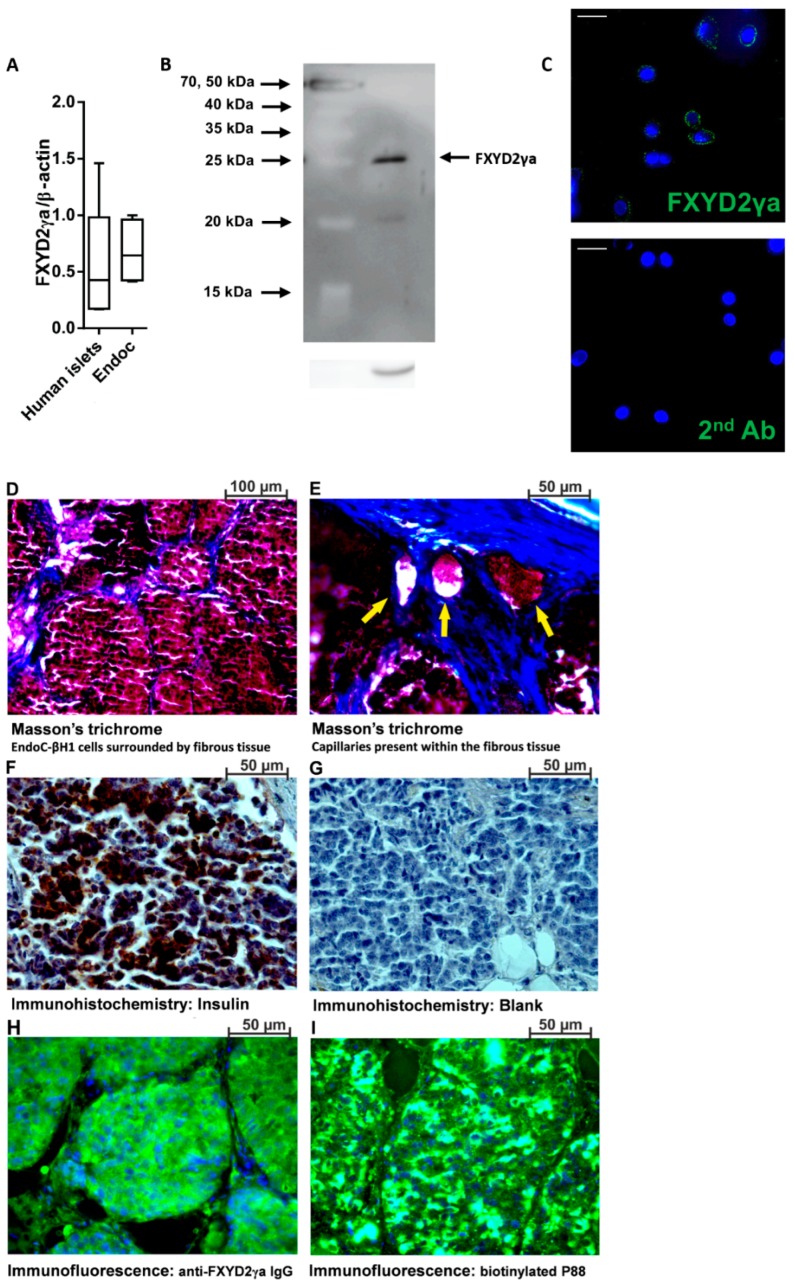
Expression of FXYD2γa in pancreatic human islets and EndoC-βH1 cells. (**A**) Quantitative RT-PCR (qPCR) of FXYD2γa mRNA expression in EndoC-βH1 cells (*n* = 5) and human pancreatic islets (*n* = 4). Data are presented as a box-plot. (**B**) A representative immunoblot of EndoC-βH1 cells, with alpha-tubulin as a reference protein (*n* = 3); (**C**) Immunocytochemistry of EndoC-βH1 cells showing surface localization of FXYD2γa (green) and Hoechst staining of nuclei (blue) (*n* = 3). The negative staining control of EndoC-βH1 cells (without the FXYD2γa antibody) is presented at the right side of panel C; the white scale bar represents 1 µm. The luminosity and the contrast were uniformly increased in both pictures to improve visualization. The original pictures are shown in Figure S5 (**D**–**I**). Histological evaluation of the implanted EndoC-βH1 tumors, showing cells clustered into pseudo-islets surrounded by fibrotic tissue (**D**), capillary networks (yellow arrows) (**E**), insulin expression (**F**) with its corresponding negative control (**G**) and FXYD2γa detected with either SPY393 polyclonal antibody (**H**) or the biotinylated P88 (**I**); (**D**–**I**) are representative micrographs from 2 EndoC-βH1 cell-implanted mice.

To investigate the ability of Gd-DOTA-P88 to enhance the MRI contrast of in vivo xenotransplanted human beta cells, we implanted mice with human insulin-producing EndoC-βH1 cells. Of note, this imaging probe targets specifically human FXYD2γa, which prevents us from imaging endogenous mouse beta cells. The mRNA expression of FXYD2γa is similar in EndoC-βH1 cells and human pancreatic islets, validating the use of the cell line in these experiments (Figure 3A). FXYD2γa protein was clearly detected in EndoC-βH1 cells (Figure 3B), where it has a cell surface localization (Figure 3C).

Nine weeks after post-transplantation, the mice developed EndoC-βH1 intra-muscular cell masses (volume of 13, 42 ± 229 mm^3^; diameter of 2.70 ± 0.07 mm), with detectable human C-peptide in the range of 26–340 pmol/L. The implanted EndoC-βH1 cells were organized into pseudo-islets (diameter between 50 and 200 µm) that were encapsulated in fibrotic tissue with an embedded capillary network (Figure 3D,E). The pseudo-islets express both insulin (Figure 3F) and FXYD2γa (Figure 3H,I).

**Figure 4:**
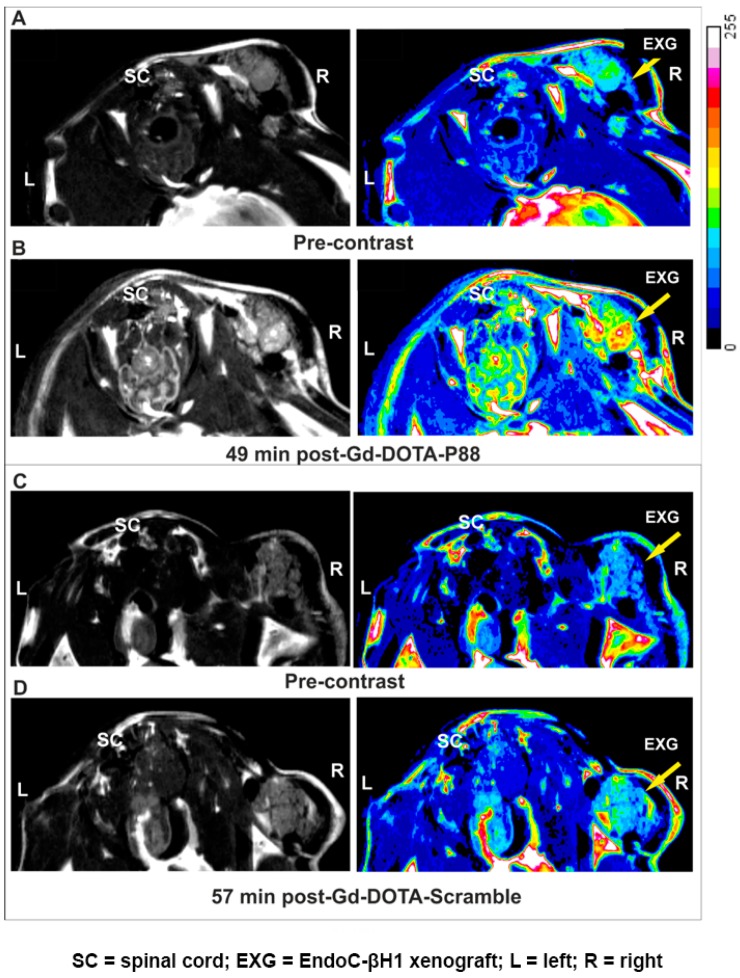
Non-invasive MRI of EndoC-βH1 xenograft bearing-mice using Gd-DOTA-P88 and Gd-DOTA-Scramble. (**A**–**D**) Representative MR images of mice bearing EndoC-βH1 transplants. Pre-contrast images (**A,C**) were acquired before injection of CAs and the post-contrast images were obtained 50 min (**B,D**) after i.v. administration of 0.1 mmoL Gd/kg b.w. of Gd-DOTA-P88 (**B**) or Gd-DOTA-Scramble (**D**). Mice were implanted with EndoC-βH1 grafts in transplantation rings in the right hind leg and vehicle transplantation rings in the left hind leg. The images are displayed in grey-scale (left row) and in pseudo-coloring overlay (right row). The images are representative of 4–6 similar experiments. Legend: SC = spinal cord; EXG = EndoC-βH1 xenograft; L = left; R = right.

**Figure 5:**
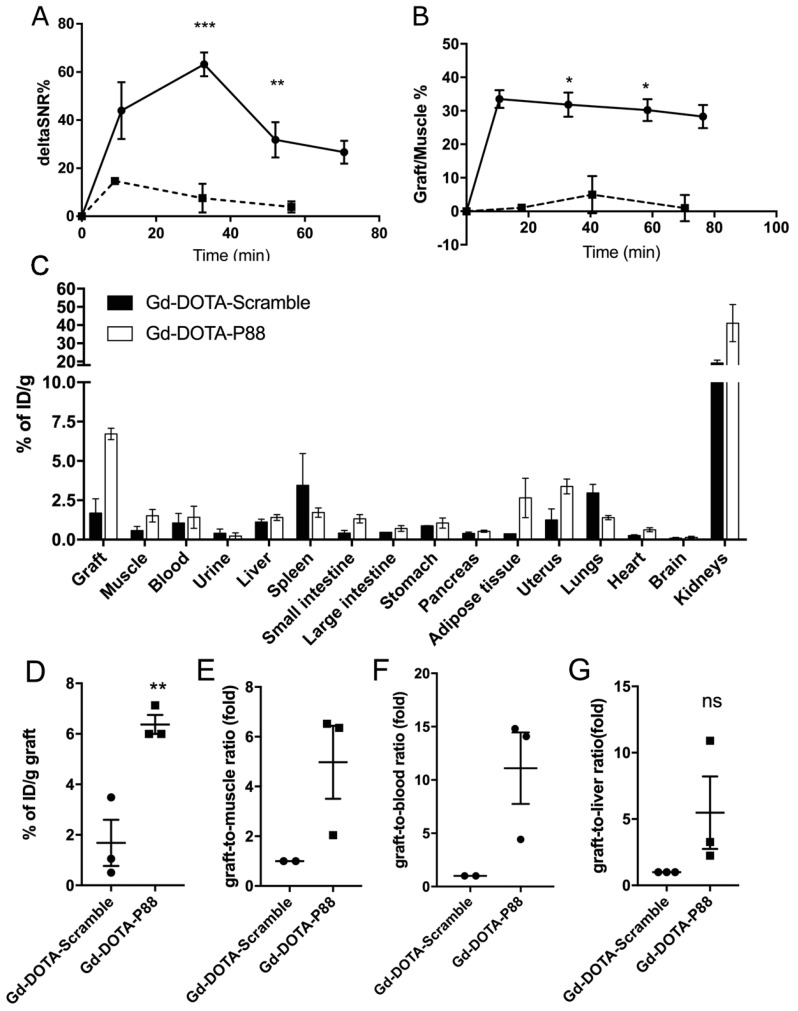
The contrast enhancement of EndoC-βH1 xenografts and the biodistribution profile of Gd-DOTA-P88 and control CA in mice implanted with EndoC-βH1 cells. (**A**,**B**) Quantification of the acquired images within regions-of-interest (ROIs) drawn around each xenograft and expressed as ΔSNR% (A) or X/M % (**B**). Data are expressed as ROI ± SD of 4–6 experiments; unpaired Student’s *t*-test; * *p* ≤ 0.05; ** *p* ≤ 0.01; *** *p* ≤ 0.001. (**C**–**G**) The biodistribution analysis of Gd-DOTA-P88 and control CA Gd-DOTA-Scramble was performed in mice xenografted with human EndoC-βH1 (*n* = 3) (**C**). The evaluation was done at 90 min after i.v. administration of 0.1 mmoL Gd/kg b.w. of Gd-DOTA-P88 (white bars) or control CA Gd-DOTA-Scramble (black bars) and expressed as percentage of the injected dose per gram of dried tissue (% of ID/g) ± SEM; (**D**–**G**)). Individual uptake levels of the CAs in EndoC-βH1 grafts (**D**, *n* = 3) and the selected background tissues, where the results are expressed as graft-to-muscle (**E**, *n* = 2), graft-to-blood (**F**, *n* = 2) and graft-to-liver (**G**, *n* = 3) ratios of individual mice. Data are presented as mean ± SEM; unpaired Student’s *t*-test, * *p* ≤ 0.05.

This in vivo model was employed to evaluate the FXYD2γa expression by a non-invasive imaging method, namely, MRI. The MR images of transplanted pseudo-islets (TPI) were obtained between 0–70 min following CA injection, while the CA biodistribution was evaluated 90 min after the i.v. administration of Gd-DOTA-P88 or the control Gd-DOTA-Scramble. Gd-DOTA-P88 accumulation in the TPI was clearly visualized by MRI (Figure 4A,B), where it produced a positive contrast originating from the EndoC-βH1 xenografts. On the other hand, Gd-DOTA-Scramble induced only a minor post-contrast enhancement (Figure 4C,D). In addition, Gd-DOTA-P88 gave no-post-contrast gain in the empty control ring (Figure S3). Based on the time-dependent acquisition and image quantification, the Gd-DOTA-P88 produced a significant T_1_-weighted post-contrast increase at 33 min for both ΔSNR% and the xenograft/muscle (X/M %) (*p* < 0.0001 and *p* < 0.01, respectively) and at 52 min post-injection (*p* < 0.05) over the control CA (Figure 5A,B).

**Figure 6:**
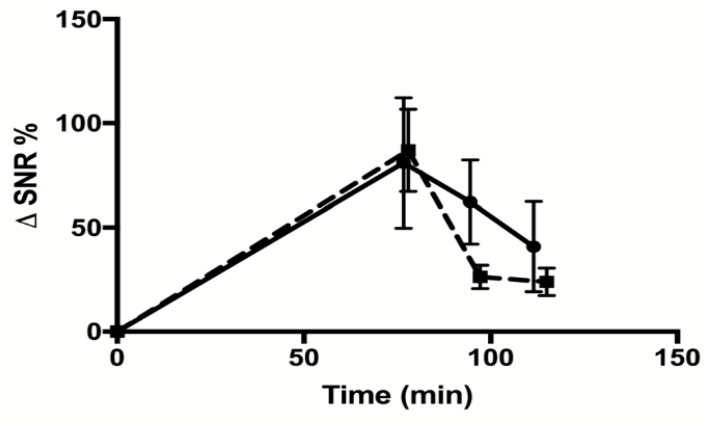
Measurement of renal clearance of Gd-DOTA-P88 and Gd-DOTA-Scramble. Mice were implanted with CHO-FXYD2γa^+^ in the right hind leg and wildtype CHO cells in the left hind leg. MRI images were taken after 0, 75, 95, and 115 min. The signals emitted by the kidneys were quantified within regions-of-interest (ROIs) drawn around each kidney. The contrast enhancement is expressed as ΔSNR%. The solid line indicates the values obtained for Gd-DOTA-P88 and the dashed line indicates those of Gd-DOTA-Scramble. The data are expressed as means ± SD, *n* = 3–4 mice in each group.

The biodistribution analysis (Figure 5C) showed that the Gd-DOTA-P88 accumulated significantly in the EndoC-βH1 xenografts as compared to the Gd-DOTA-Scramble (*p* < 0.05) (Figure 5D). Both CAs showed high uptake in the kidneys, with a fast elimination of unbound CA from blood (i.e., 1.4% of ID/g for Gd-DOTA-P88; 1.6% of ID/g for Gd-DOTA-Scramble) and non-targeted tissues at 75–80 min after administration; this is the usual pattern of renal excretion of paramagnetic CAs. The ratios for Gd-DOTA-P88 accumulation in EndoC-βH1 xenografts as compared to key background tissues were as follows: xenograft-to-muscle, xenograft-to-blood and xenograft-to-liver ratios respectively of 4.3 ± 2.4, 4.6 ± 3.4 and 4.9 ± 0.8, with a significant higher xenograft-to-liver ratio in comparison to the Gd-DOTA-Scramble (*p* < 0.05) (Figure 5E–G). In order to exclude an artificial accumulation of Gd-DOTA-P88 due to a reduced renal clearance, we compared the ΔSNR% measured in kidneys for both probes. The clearance kinetics were similar for the two CAs (Figure 6).

**Figure 7:**
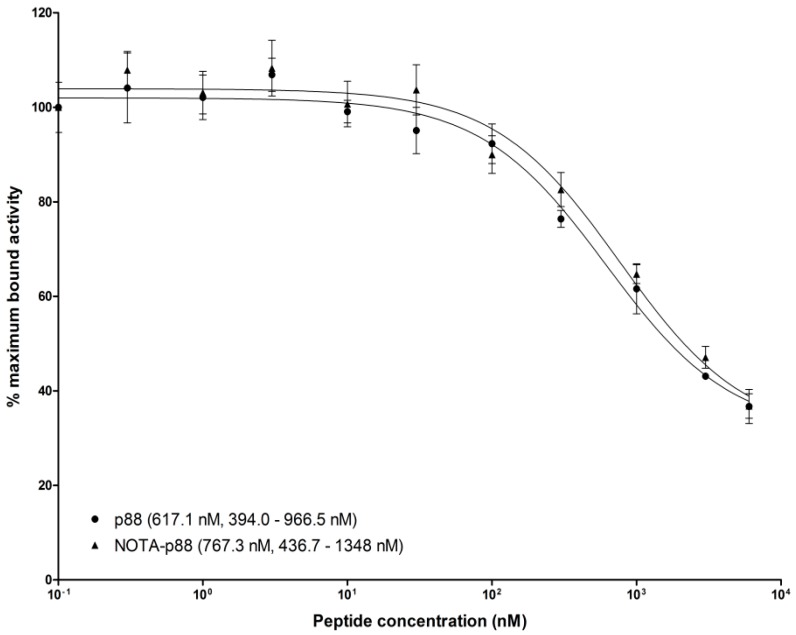
Competitive binding assay. CHO-FXYD2 cells were seeded in six-well plates at a density of 10^6^ cells/well 24 h prior to the experiments. Serial dilutions of unlabeled NOTA-P88 and P88, at a final concentration ranging from 0.1 to 6000 nM (*n* = 3), were added along with 1000 Bq [^111^In]In-NOTA-P88. After 4 h incubation at 37 °C, the cells were washed, and the cell-associated radioactivity was determined in a well-type γ-counter. The IC_50_ values were calculated by one-site competition analysis.

To determine the binding affinity of P88, we performed an in vitro competitive binding assay. For this purpose, NOTA-P88 was labeled with a specific activity of 15 MBq/nmol, reaching a radiochemical purity of ~90%. The IC_50_ value for NOTA-P88 was 767.3 nM, which did not differ significantly from the IC_50_ value of unconjugated P88 (617.1 nM) (Figure 7).

## 3. Discussion

Aiming to observe the human insulin secreting cells in vivo, we have developed and validated a novel paramagnetic CA based on a peptide (P88) that targets the beta cell biomarker FXYD2γa [13]. For this purpose, P88 was conjugated to a Gd-DOTA complex, generating Gd-DOTA-P88. Its utility for in vivo use was evaluated by MRI and biodistribution in immunodeficient mice transplanted with human insulin-producing EndoC-βH1 cells. The data obtained show that Gd-DOTA-P88 enables in vivo imaging of human insulin-secreting cells or human islet grafts.

Imaging of BCM in both pre-clinical and clinical settings is a long-sought goal [4], requiring the discovery of both beta cell specific biomarkers and adequate tracers targeting them. FXYD2γa ([12] and present data) is specifically expressed at the cell membrane of human beta cells and is not affected by exposure to inflammatory mediators [12]. It is thus a potentially interesting biomarker for BCM estimation under the pro-inflammatory conditions to which islet cells are exposed in T1D or following islet transplantation. The P88 functionalized CA, specifically binding FXYD2γa, is thus an interesting candidate for imaging of human insulin secreting cells.

The present MRI and biodistribution data indicate that Gd-DOTA-P88 has xenograft-to-liver and xenograft-to-muscle ratios adequate for the discrimination of intra-hepatic or intra-muscular transplanted human insulin secreting cells from the background tissues. This suggests that imaging of the transplanted pancreatic islets or beta cells is feasible and would allow the non-invasive and in situ monitoring of their outcome. The intra-muscular pseudo-islets developed by transplanted EndoC-βH1 cells reached a mean diameter of about 2.7 ± 0.07 mm (volume of about 13.42 ± 2.29 mm^3^), which is similar to that of human islet grafts (about 2.4 mm) [19], indicating that our mouse model simulates this clinical condition. Moreover, the mean diameter (125 µm) and volume (0.000785 mm^3^ or 0.000785 µL) of EndoC-βH1 pseudo-islets reproduce the mean diameter (110 µm) and volume (0.00068 mm^3^ or 0.00068 µL) of human pancreatic islets [20]. An islet-like structure could comprise about 444 EndoC-βH1 cells (for a cell diameter of 15 µm and volume of 1.77 × 10^−6^ mm^3^) or about 254 beta cells (57.13%) in the case of human pancreatic islets [20]. If each one expresses about 10^−15^ mol of FXYD2γa [10], it corresponds to 4.44 × 10^−13^ mol of FXYD2γa per EndoC-βH1 islet (or 2.55 × 10^−13^ mol per human islet) and a concentration of 566 µM (or 325 µM for human islets). This concentration would be more than sufficient to allow detection of an islet graft of 13.42 mm^3^ by MRI using an in-plane resolution of 107 × 117 µm (voxel of 0.0014 mm^3^) like in our case (at 7 T) or of 200 × 200 µm (voxel of 0.008 mm^3^) obtained in clinical conditions at 1.5 T [19].

Importantly, Gd-DOTA-P88 is almost entirely cleared from the blood and non-targeted tissues after 75–80 min post injection, while it remains bound to the target beta cells, which may favor specific imaging. As shown by MRI and biodistribution data, the pattern of renal clearance of both Gd-DOTA-P88 and Gd-DOTA-Scramble is not significantly different, which suggests that xenograft enhancement by the specific compound is not a consequence of its delayed blood clearance. On the other hand, Gd-DOTA-P88 is less efficient in terms of NMR efficacy (i.e., relaxivity: r_1_ of 4.57 s^−1^ mM^−1^ at 300 MHz and 37 °C for Gd-DOTA-P88) than superparamagnetic agents (i.e., r_2_ of 92.93 s^−1^ mM^−1^ at 300 MHz and 37 °C for USPIO-P88 [14,15]), but the binding of functionalized paramagnetic compounds to their targeted biomarkers contributes to a substantial enhancement of their relaxivity due to the increase in the rotational correlation time [21,22]. Finally, the binding specificity of P88 was evaluated by using an in vitro competitive assay. The IC_50_ was comparable for both the unconjugated probe and NOTA-P88, suggesting that the conjugation of a chelator moiety does not interfere with its binding properties.

Paramagnetic imaging probes are already approved for clinical applications, and MRI scanners are largely available. Thus, the presently developed Gd-DOTA-P88 might become a valuable tool for future clinical translation, more especially for the in vivo quantification of intra-muscular or intra-hepatic human islet grafts. The present work, combined with our previous studies [12,13], increases the robustness and applicability of this CA and the translational potential of the targeted biomarker.

## 4. Materials and Methods

### 4.1. Ethical Statements

Pancreases not suitable for clinical purposes were obtained with informed written consent from anonymized organ donors [23]. All the experiments and methods using human pancreatic islets were approved by and performed in accordance with the guidelines and regulations of the regional ethics committee of the Pisa University, Italy and in line with the 1964 Helsinki declaration and its later amendments or comparable ethical standards. Human islet isolation and culture was performed as previously described [24]. The human pancreatic islets or pancreas preparations used in the present study are described in Table S1.

Six-week old male NMRI-*Foxn1^nu^/Foxn1^nu^* or female SCID CB-17/Icr-*Prkdc^scid^*/Rj mice (Janvier Labs, St. Berthevin, France) were used and housed in accordance with the Animal Act 1986/2013, Belgium. The experiments were approved by the Ethical Committee for Animal Welfare (CEBEA) of the ULB, University of Mons, and ULB-Center for Microscopy and Molecular Imaging (CMMI) (Campus Biopole Charleroi, Charleroi, Belgium) (ethical permits CMMI-2013-03;2014, 485N and MU/02/03), Belgium. All applicable institutional and/or national guidelines for the care and use of animals were followed.

### 4.2. Cell Culture and Transfection

Human insulin-producing EndoC-βH1 cells [25] and human pancreatic islets were cultured as described [26]. CHO cells were cultured at 37 °C, 5% CO_2_ in RPMI 1640 media supplemented with 2 mM GlutaMAX and 10% FBS (all from Invitrogen, Gent, Belgium). To generate FXYD2γa overexpressing cells, the CHO cells were transfected with a human FXYD2γa plasmid (#RC205076, Origene, Rockville, MD, USA) together with Lipofectamine 2000 as transfection agent according to the manufacturer’s instructions (Invitrogen, Carlsbad, CA, USA). The cells where thereafter selected with Geneticin-G418 (50 μg/mL) (Invitrogen, Carlsbad, CA, USA). The mRNA expression of FXYD2γa was quantified using primers for the human FXYD2γ variant a and beta actin as in [12]. The gene copy number measured in the transfected cells was compared with the CT [27] of beta actin, used as a housekeeping gene.

### 4.3. Immunoblotting and Immunocy to Fluorescence

Immunoblot analyses were performed with 10^5^ EndoC-βH1 cells on a 14% polyacrylamide gel as in [26] with a rabbit anti-FXYD2γa polyclonal antibody (pAb) designated SPY393 (7.5 µg/mL) (Eurogentec, Seraing, Belgium). Alpha-tubulin, detected by a monoclonal antibody (mAb) (1:5000; #T5168; Sigma-Aldrich, Overijse Belgium), was used as a reference (“housekeeping”) protein. Detection and the quantification were performed as described [26].

EndoC-βH1 cells were stained for 1 h on ice with the same pAb (1:200; SPY393) without any prior fixation or permeabilization. An anti-rabbit Alexa fluor 480 nm conjugated secondary antibody (ThermoFisher Scientific, Gent, Belgium) was applied and the slides were imaged as described [26].

### 4.4. Mouse Models Implanted with Human EndoC-βH1 or CHO-FXYD2γa^+^ Cells

CHO-FXYD2γa^+^ and wild type CHO cells were implanted in female SCID CB-17/Icr-*Prkdc^scid^*/Rj mice by s.c. injections in the right and left hind legs, respectively. 3 × 10^6^ CHO cells pre-mixed 1:1 (*v/v*) with Matrigel (Corning, Lasne, Belgium) were injected per site.

Male NMRI-*Foxn1^nu^/Foxn1^nu^* and female SCID CB-17/Icr-*Prkdc^scid^*/Rj mice were implanted with human insulin-producing EndoC-βH1 cells as described [25,28]. Male NMRI-*Foxn1^nu^/Foxn1^nu^* mice were used for methodological development/validation, while follow up experiments were performed in female SCID CB-17 mice. On the day of inoculation, 4–6 × 10^6^ EndoC-βH1 cells were seeded on a rubber toric joint (EFJM, Saint Lubin des Joncherets, France), supported in Matrigel HC (Corning, New York, NY, USA) supplemented with MmVEGF-164 (1 ng/mL) (BioLegend, San Diego, CA, USA). The cell-containing or the empty vehicle rubber rings were then inserted under the epimysium in the biceps or quadriceps femoris muscle. The mice were anesthetized with 3% isoflurane and received short-term analgesic (buprenorphine 0.1 mg/kg) and then long-term analgesic (3 mg/mL of acetaminophen-supplemented water for 10 consecutive days) respectively before and after the surgery. Random glycaemia was measured weekly with an ACCU-CHEK Nano glucometer (Roche, Brussels, Belgium). Once the cell xenograft became palpable, the mice received 20% glucose-supplemented drinking water to counter the progressive hypoglycemia induced by the EndoC-βH1 cells. Human C-peptide was measured in plasma with a human Ultrasensitive C-peptide ELISA (Mercodia, Uppsala, Sweden). This is a pilot study, with an arbitrarily selected group of animals.

### 4.5. Validation of Cell Xenografts by Histochemistry, Immunohistochemistry and Immunofluorescence

For histological evaluation, the mice were euthanized with a lethal dose (600 mg/kg b.w., i.p.) of Pentobarbital (Sanofi, Brussels, Belgium) and the xenografts were sampled after transcardiac perfusion with phosphate buffered saline (PBS, Arlington, VA, USA). The xenografts were fixed in 4% paraformaldehyde, followed by dehydration and paraffin embedding. Histological staining was performed on 5 µm sections, which were dewaxed and rehydrated. The Masson’s trichrome stain was performed using the Accustain^®^ kit (Sigma-Aldrich, St. Louis, MO, USA) according to the manufacturer’s instructions. Insulin and FXYD2γa expression and the binding of biotinylated P88 were evaluated as described [11]. Briefly, insulin was detected with 2 µg/mL of anti-insulin mouse mAb (Abcam, Cambridge, UK), followed by a peroxidase conjugated antibody (Sigma-Aldrich, St. Louis, MO, USA) and stained with 3, 3′-diaminobenzidine (DAB) tetrachlorhydrate (Sigma-Aldrich, St. Louis, MO, USA) and 0.02% H_2_O_2_. For FXYD2γa detection, addition of the rabbit pAb SPY393 (12.5 µg/mL; Eurogentec) was followed by fluorescein goat anti-rabbit IgG (20 μg/mL; Vector Labconsult, Brussels, Belgium). The biotinylated P88 (10 µM) was detected with 10 µg/mL of goat anti-biotin antibody, followed by fluorescein rabbit anti-goat (20 µg/mL) antibody (both from Vector Labconsult, Brussels, Belgium), and Vectashield mounting medium with DAPI. The microphotographs were acquired on a DM2000 Leica microscope equipped with a Leica DFC 425C camera (Leica Microsystems, Groot Bijgaarden, Belgium).

### 4.6. Synthesis of Gd-DOTA-P88 and Gd-DOTA-Scramble

P88 and its control peptide (Scramble) were produced by Eurogentec (Seraing, Belgium). The CAs were synthesized (Figure 1) and prepared as follows: 490 mg of peptides (0.17 mmoL) were dissolved in 15 mL of dry dimethylformamide (DMF) at room temperature under a nitrogen atmosphere. Thereafter, two batches of 95 mg (0.2 mmoL) of DOTA-Ga anhydride (Chematec, Dijon, France) were added and the solution was stirred for 36 and 24 h after each addition. The DOTA-peptide reaction was monitored by liquid chromatography–mass spectrometry (LC-MS) (Waters, Asse, Belgium) (Figure 1). The reaction was then quenched with water and dialyzed (membrane range 100 to 500 Da) (VWR, Leuven, Belgium). After 4 days of dialysis to remove unreacted DOTA-Ga, the solution was freeze-dried to obtain 520 mg of DOTA-P88 or DOTA-Scramble with a yield of approximately 90%, as determined by weighing. Mass spectrometry confirmed the molecular weight of the compounds. ESI–MS QTOF (ElectroSpray Ionization-Mass Spectrometry-Quadrupole Time of Flight) were: *m*/*z* 1687 (M + 2H)^2+^, 1698 (M + H + Na)^2+^, 1709 (M + 2 Na)^2+^ for DOTA-P88 and 1698 (M + H + Na)^2+^, 1709 (M + 2Na)^2+^, 1720 (M + 3 Na)^2+^ for DOTA-Scramble. For comparison: ESI-MS QTOF: *m*/*z* 580 (M + Na)^+^ for Gd-DOTA.

The DOTA ligands were thereafter treated with GdCl_3_ to generate the corresponding paramagnetic complexes for the MRI studies by dissolving 698 mg of DOTA-P88 in 23 mL (or 139.6 mg of DOTA-Scramble in 5 mL) of water and complexed with 77 mg (or 15 mg for DOTA-Scramble) of GdCl_3_.6H_2_O dissolved in 1 mL of water overnight at pH 6. The absence of free Gd ions was verified with Arzenazo test [29]. ESI–MS QTOF were: *m*/*z* 1176.5 (M + 3H)^3+^ 882.5 (M + 4H)^4+^ for Gd-DOTA-P88 and 1176.5 (M + 3H)^3+^ for Gd-DOTA-Scramble. The Gd-concentration in CA solutions was determined by relaxometry on a 20 MHz (0.47T) Bruker Minispec mq 20 (Bruker, Karlsruhe, Germany). The longitudinal and transverse relaxivities, r_1_ and r_2_, of Gd-DOTA-P88 (r_1_ of 4.57 s^−1^ mM^−1^; r_2_ of 6.60 s^−1^ mM^−1^) and Gd-DOTA-Scramble (r_1_ of 4.87 s^−1^ mM^−1^; r_2_ of 6.57 s^−1^ mM^−1^) were measured on a 300 MHz (7T) Bruker Biospec imaging system (Bruker, Ettlingen, Germany) equipped with a Pharmascan horizontal magnet.

### 4.7. MRI and Ex Vivo Biodistribution of Gd-DOTA-P88 and Gd-DOTA-Scramble

The MRI acquisitions were performed on a 300 MHz (7T) Bruker Biospec imaging system before and after the i.v. administration (in the caudal vein of the mice) of 0.1 mmoL Gd/kg of Gd-DOTA-P88 (*n* = 6) and Gd-DOTA-Scramble (*n* = 4). The MRI images were acquired with a rapid acquisition with relaxation enhancement (RARE) imaging sequence (CHO model: TR/TE = 463.5/33.8 ms, RARE factor = 4, NEX = 20, matrix = 256 × 256, FOV = 4 × 3.5 cm, slice thickness = 1.5 mm, 6 sagittal slices, spatial resolution = 156 × 137 µm; EndoC-βH1 model: TR/TE = 557.4/40.8 ms, RARE factor = 4, NEX = 20, matrix = 372 × 256, FOV = 4 × 3 cm, slice thickness = 1 mm, 6 axial slices, spatial resolution = 107 × 117 µm). Before each new MRI experiment, the signal was adjusted using a standardized Bruker reference comprising a solution of 1 g/L of CuSO_4_ and 4.31 g/L of NaCl in double-distilled water. The signal was then adjusted again on each new animal before starting the MRI experiment; to standardize the signal calibration, an internal reference (0.05 mM Gd-DTPA in 2% gelatine) was included at the side of each animal.

A region of interest (ROI) was drawn around the xenograft images by using the ImageJ image analysis software (National Institutes of Health, Bethesda, MD, USA) and used to measure the signal intensity (SI) (Figure S1). An ROI drawn outside of the animal’s image was used to measure the standard deviation (SD) of the noise (Noise SD). The enhancement of signal-to-noise ratio (ΔSNR%) on post-contrast images was calculated according to the following equation:

$$\Delta \mathrm{SNR}\%=\frac{\left({\mathrm{SI}}_{\mathrm{post}}/\mathrm{Noise}\;\mathrm{SD}\right)-\left({\mathrm{SI}}_{\mathrm{pre}}/\mathrm{Noise}\;\mathrm{SD}\right)}{\left({\mathrm{SI}}_{\mathrm{pre}}/\mathrm{Noise}\;\mathrm{SD}\right)}\times 100$$

where: SI_post_ = post-contrast signal SI, and SI_pre_ = pre-contrast SI.

The % SI enhancement of xenografts (X), as compared to muscle (M) on post-contrast images was calculated as follows:

$$X/M\%=\frac{\left({\mathrm{SI}}_{\mathrm{post}}^{X}/{\mathrm{SI}}_{\mathrm{post}}^{M}\right)-\left({\mathrm{SI}}_{\mathrm{pre}}^{X}/{\mathrm{SI}}_{\mathrm{pre}}^{M}\right)}{\left({\mathrm{SI}}_{\mathrm{post}}^{X}/{\mathrm{SI}}_{\mathrm{post}}^{M}\right)}\times 100$$

The mice were euthanized after the MRI acquisitions, at 90 min post-injection, and tissues (EndoC-βH1-derived tumor, muscle, liver, spleen, small and large intestine, stomach, pancreas, adipose tissue, uterus, lungs, heart, brain and kidney), blood and urine were collected from the EndoC-βH1 model. The tissues were dried for 48 h and weighed to prepare them for the subsequent microwave mineralization with a MSL-1200 Mega unit (Milestone Inc., Sorisole, Italy) as in [30]. After dissolution, the samples were evaporated and re-dissolved in 4 mL of 5% HNO_3_. For the measurement of the Gd concentration, an aliquot (depending on the initial size of the sample) was diluted in 5 mL of 5% HNO_3_. The Gd concentration of the samples was then measured with a quadrupole inductively coupled plasma mass spectrometry (ICP-MS) Agilent 7700 (Agilent Technologies Inc., Diegem, Belgium), where Indium was used as internal standard for correcting the instrumental drift, and the internal total reproducibility was controlled with both synthetic and natural standards and was below 10% RSEM.

### 4.8. Synthesis of NOTA-P88 and Radiolabeling

NOTA-P88 was synthesized as followed. First, the *n* peptide (0.2 g, 0.07 mmoL) was dissolved in 15 mL of dry DMF at room temperature under nitrogen atmosphere. NODA-GA(*t*-butyl)_3_ (40 mg, 0.072 mmoL), DEPBT (3-(diethoxyphosphoryloxy)-1,2,3-benzotriazin-4(3H)-one, 40 mg, 0.15 mmoL) and DIPEA (*N*,*N*-Diisopropylethylamine, 36 µL, 0.27 mmoL) were added to the semi-gel solution of peptide. A volume of 5 mL of dimethylformamide (DMF) was added to break the gel and stirred for 36 h. Reaction was monitored by LC-MS. A quantity of 0.02 mmoL of NODAGA was added to this mixture after 36 h and stirred for another 24 h for completion of the peptide. The reaction mixture was quenched with a few drops of water, and then, the compound was precipitated with 50 mL of diethylether. The resultant precipitate was dried and washed well with diethylether. The product was purified by reverse phase flash chromatography. The purified product was mixed to 20% TFA, a catalytic amount of TIPS was added in dichloromethane and stirred for 10 h. The reaction completion was monitored by LC-MS. The product was precipitated in diethylether, washed with ether and dissolved in water. The aqueous medium was freeze dried. This process was repeated several times to remove excess trapped TFA (Figure S4). The product was characterized by ESI-MS QTOF: calculated for C_144_H_214_N_42_O_45_S is 3285 dalton found: 1709.5 (M + 2H + 3 Na)^2+^, 1643.5 (M + 2H)^2+^, 1140 (M + 3H +2 Na)^3+^, 1096 (M + 3H)^3+^.

NOTA-P88 was labeled with ^111^In by adding together two volumes of 0.5 M MES buffer, pH 5.5, one volume of ^111^InCl_3_ and 1 µg of peptide. After incubation at room temperature for 15 min, 50 mM EDTA (ethylenediaminetatreacetic acid) (Sigma-Aldrich, St. Louis, MO, USA) was added to a final concentration of 5 mM to chelate any free ^111^InCl_3_. Additionally, 10% Tween-80 (Sigma-Aldrich) in PBS was added to a final concentration of 0.1% to avoid adhesion of the peptide to the reaction vial. Radiochemical purity was determined by instant thin layer chromatography on silica-gel strips (ITLC-SG Biodex, Shirley, NY, USA). As a mobile phase 0.1 M EDTA in 0.1 M NH_4_Ac was used; Rf ^111^In-NOTA-p88 = 0, Rf ^111^In-EDTA = 1.

### 4.9. Competitive Binding Assay

The 50% inhibitory concentrations (IC_50_) of P88 and NOTA-P88 were determined in a competitive binding assay using CHO-FXYD2 cells. Cells were seeded in 6-well plates at a density of 1 × 10^6^ cells/well 24 h prior the experiment. [^111^In]In-NOTA-P88 was used as tracer and was labeled as described above. Cells were washed with HAM-F12 supplemented with 0.5% *w*/*v* BSA (bovine serum albumin). Serial dilutions of unlabeled NOTA-P88 and P88, at a final concentration ranging from 0.1 to 6000 nM (*n* = 3), were added along with 1000 Bq [^111^In]In-NOTA-P88. After 4 h incubation at 37 °C, the cells were washed as described above and were harvested with 0.1 M NaOH. The cell-associated radioactivity was determined in a well-type γ-counter (Wallac 1480-Wizard, Perkin Elmer, Boston, MA, USA). The IC_50_ values were calculated by one-site competition analysis using GraphPad Prism (version 5.03, GraphPad Software, San Diego, CA, USA).

### 4.10. Statistical Analyses

Data are presented as means ± SEM or SD and plotted as scatter or box plots, indicating lower quartile, median, and higher quartile, with whiskers representing the range of the remaining data points. Comparisons were performed by two-tailed paired or unpaired Student’s *t*-test or by ANOVA followed by Student’s *t*-test with Šídák correction, as indicated, using Graph Pad Prism 6 software (Graph Software Inc., San Diego, CA, USA). The data from the competitive binding assay were analyzed using GraphPad Prism software version 5.03 for Windows. The F-test was used to manually calculate significance. In all cases, a *p*-value below 0.05 was considered significant.

## 5. Conclusions

The present work shows that the Gd-DOTA-P88 is a suitable CA to non-invasively image human insulin secreting cells implanted into immunodeficient mice. Pending additional work, this new tracer may become a useful tool to pre-clinically quantify human islet grafts.

## Supplementary Materials

The following are available online. Table S1: Clinical characteristics of the organ donors used for human islet isolation, Figure S1: MR imaging using Gd-DOTA-P88 and Gd-DOTA-Scramble in mice implanted with CHO-FXYD2γa+ or wildtype CHO cells, Figure S2: Confirmation of FXYD2γa expression in CHO cells transfected with the plasmid encoding FXYD2γa, Figure S3: Non-invasive MR imaging of EndoC-βH1 tumors in mice using Gd-DOTA-P88, Figure S4: Coupling of P88 peptide to NOTA, Figure S5: original pictures for Figure 3B,C.
