# Supplementary material for: Imaging of Human Insulin Secreting Cells with Gd-DOTA-P88, a Paramagnetic Contrast Agent Targeting the Beta Cell Biomarker FXYD2γa
Source: Molecules. 2018 Aug 21;23(9):2100. doi: 10.3390/molecules23092100 (PMC6225257; doi:10.3390/molecules23092100)
Supplement: Supplementary file 1 [file molecules-23-02100-s001.pdf]

Supplementary information for:

Imaging of human insulin secreting cells with Gd-DOTA-P88, a paramagnetic contrast agent targeting the beta cell biomarker FXYD2γa

Stéphane Demine[@1*], Alexander Balhuizen[1*], Vinciane Debaille[2], Lieke Joosten[3], Maïté Fereau[4], Satya Narayana Murth Chilla[4], Isabelle Millard[1], Raphaël Scharfmann[5], Dominique Egrise[6], Serge Goldman[6], Piero Marchetti[7], Martin Gotthardt[3], Sophie Laurent[4,6], Carmen Burtea[4], Decio L. Eizirik[1]

[1]ULB-Center for Diabetes Research, Medical Faculty, Université Libre de Bruxelles (ULB), Route de Lennik 808-CP618, 1070, Brussels, Belgium.

[2]Laboratoire G-Time, Université Libre de Bruxelles (ULB), Av. F.D. Roosevelt 50 CP 160/02, 1050 Brussels, Belgium.

[3]Department of Radiology and Nuclear Medicine, Radboud University Medical Center, Nijmegen, The Netherlands.

[4]Department of General, Organic and Biomedical Chemistry, NMR and Molecular Imaging Laboratory, University of Mons, Avenue Maistriau 19, Mendeleev Building, B-7000 Mons, Belgium.

[5]INSERM U1016, Université Paris-Descartes, Institut Cochin, Paris, France.

[6]Center for Microscopy and Molecular Imaging (CMMI), Université Libre de Bruxelles (ULB) and University of Mons, 12 rue des professeurs Jeener et Brachet, 6041 Charleroi-Gosselies, Belgium

[7]Department of Clinical and Experimental Medicine, and University Hospital, University of Pisa, Pisa, Italy.

*= These authors contributed equally to this work.

Supplementary Table 1: Clinical characteristics of the organ donors used for human islet isolation.

Sample identity	Gender	Age (years)	BMI (Kg/m^2)	Purity (% beta cells)	Cause of Death
ID291108	Female	77	23.8	45	Trauma
ID151214	Male	79	25.3	48	Cerebral hemorrhage
ID110215	Male	44	27.7	59	Post-anoxic encephalopathy
ID200810	Male	59	24.9	70	Trauma
ID290110	Female	74	27.1	38	Unknown

Isolated human islets were used for quantitative PCR (qPCR). All donors were anonymized and the material was annotated with a sample identity. The % of beta cells in each preparation was defined by insulin immunofluorescence.

Figure S1

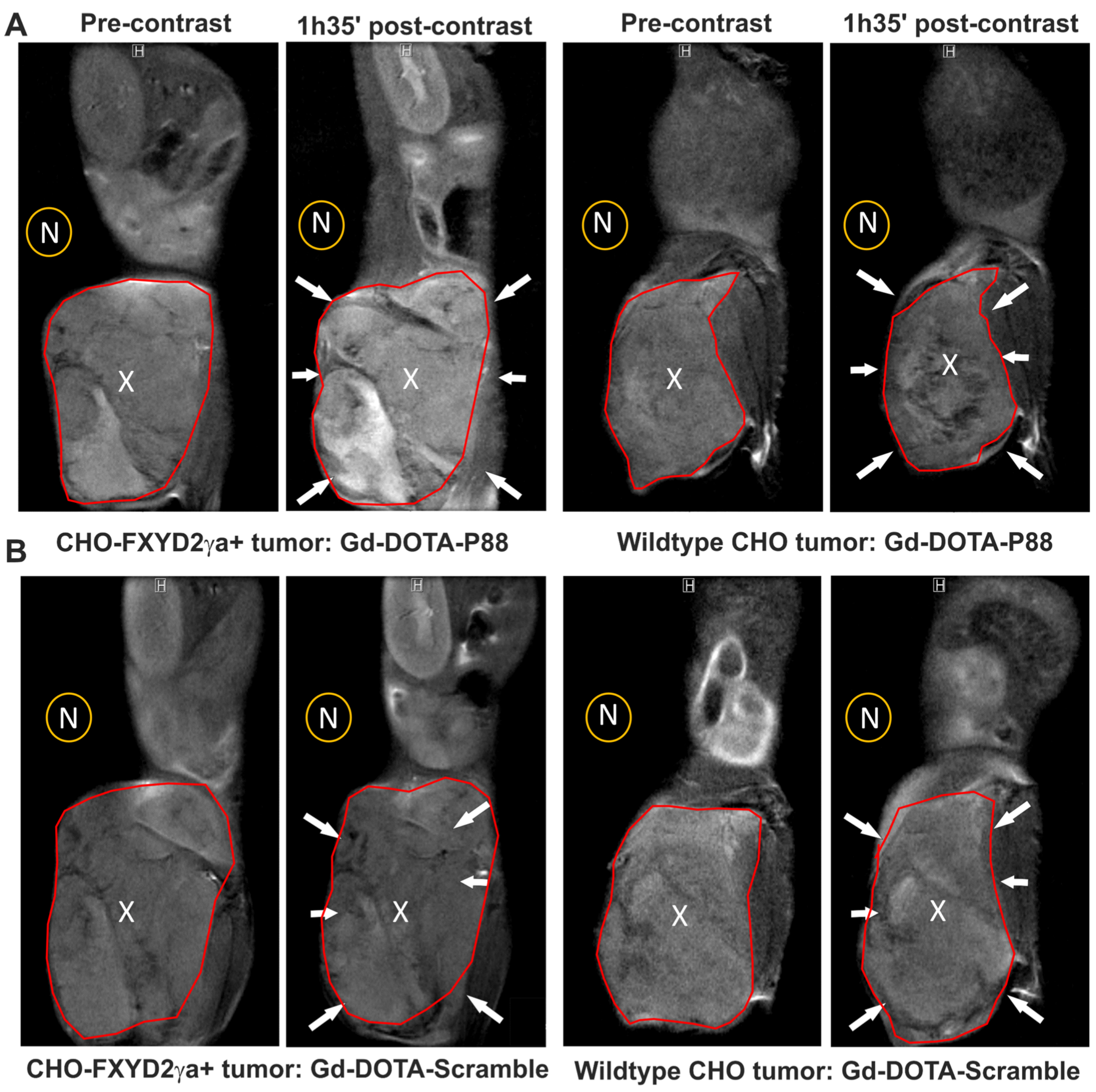
A
Pre-contrast
1h35' post-contrast
Pre-contrast
1h35' post-contrast
N
X
CHO-FXYD2γa+ tumor: Gd-DOTA-P88
Wildtype CHO tumor: Gd-DOTA-P88
B
CHO-FXYD2γa+ tumor: Gd-DOTA-Scramble
Wildtype CHO tumor: Gd-DOTA-Scramble

Figure S2

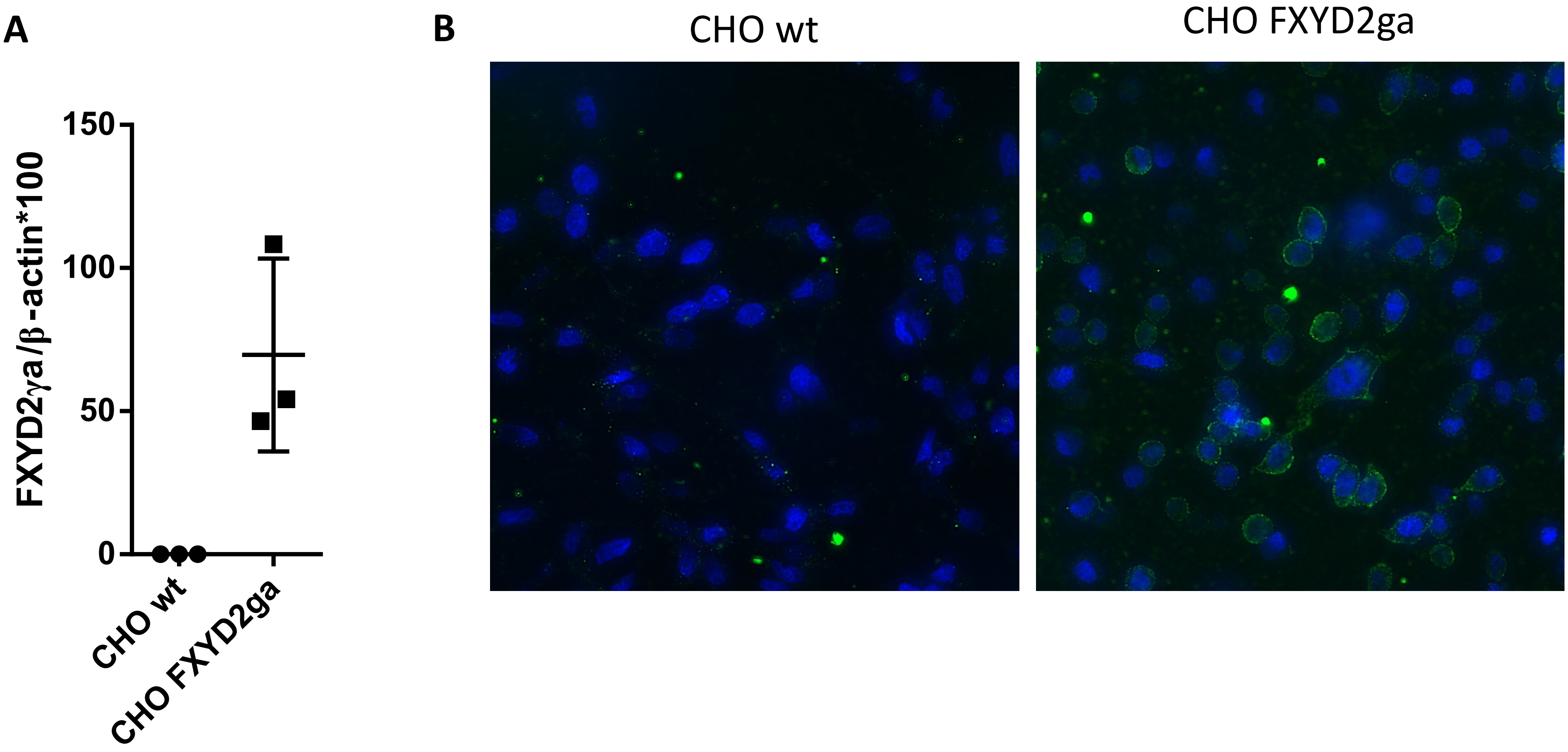

A
B
CHO wt
CHO FXYD2ga
150
100
50
0
FXYD2γa/β-actin*100
CHO wt
CHO FXYD2ga

Figure S3

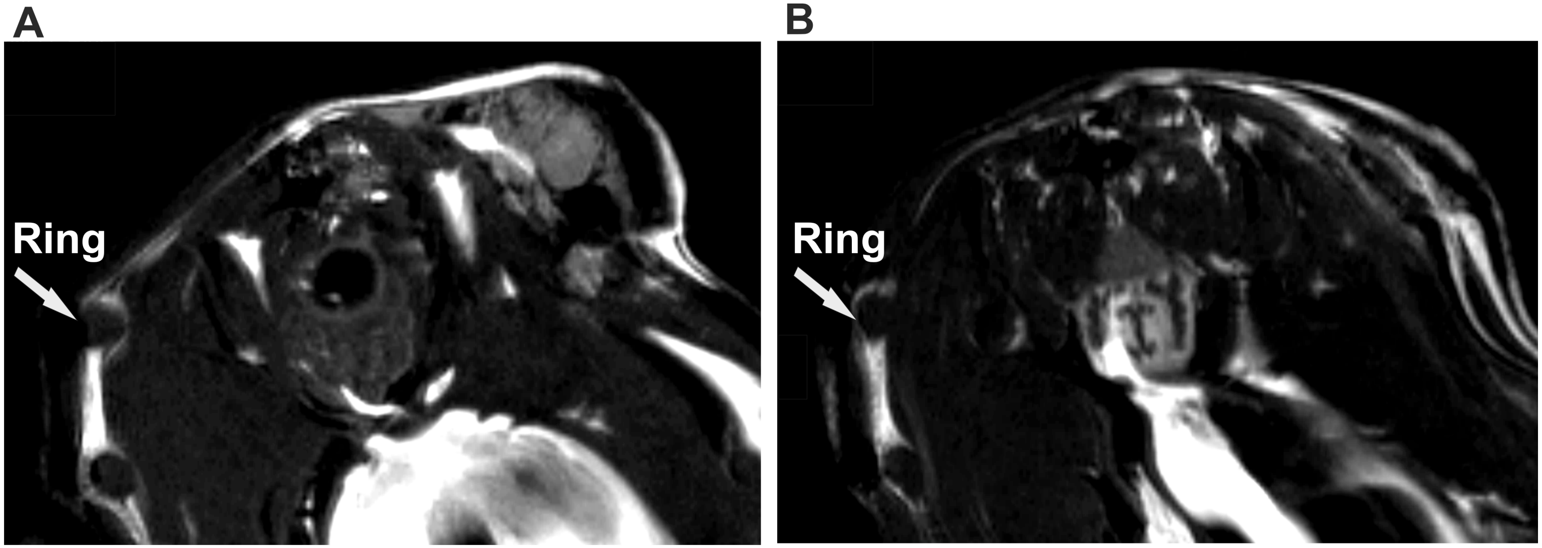

Pre-contrast

49 min post-Gd-DOTA-P88

Figure S4

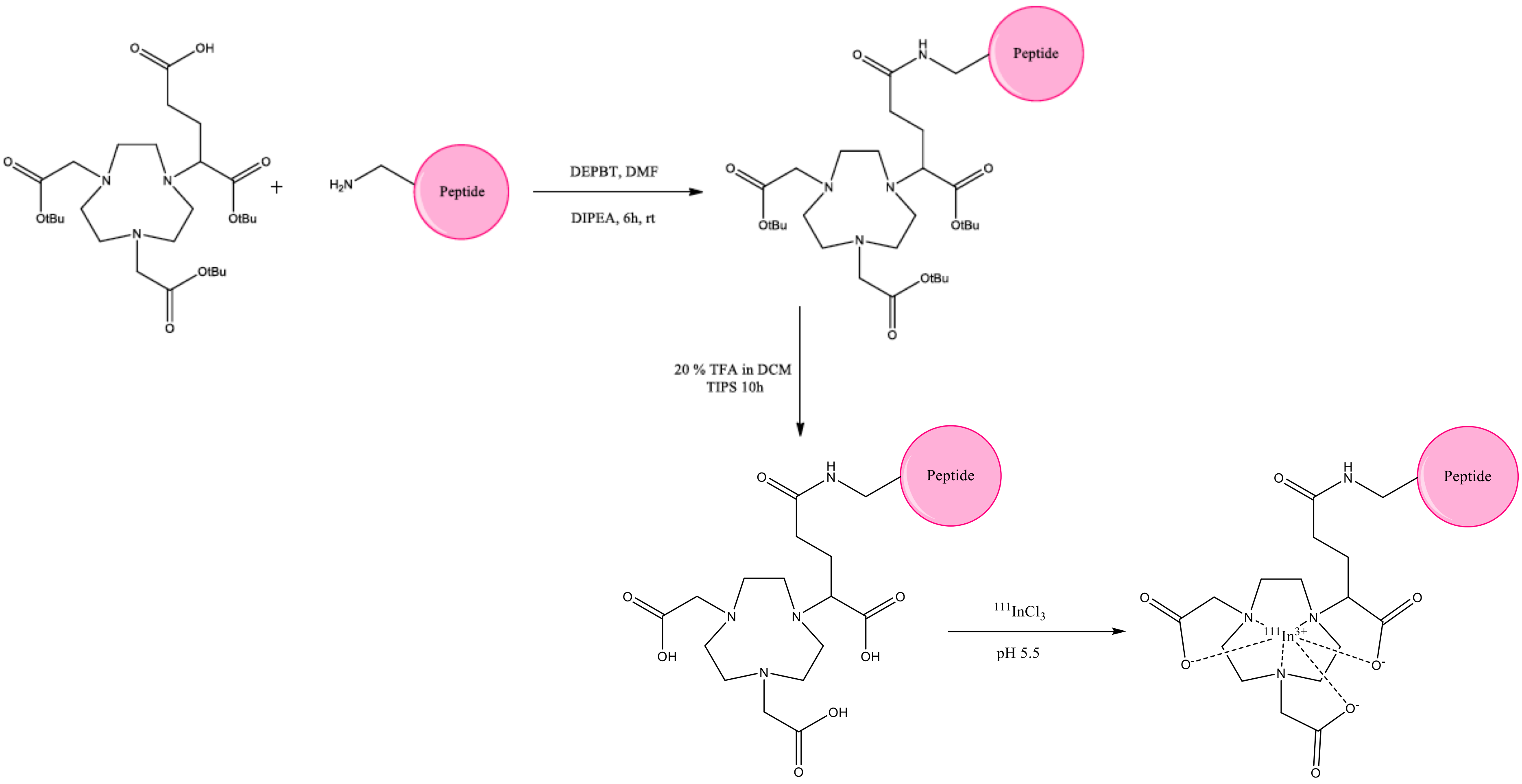

OH
O
O
N
N
O
OtBu
OtBu
N
OtBu
O
+
H2N
Peptide
DEPBT, DMF
DIPEA, 6h, rt
20 % TFA in DCM
TIPS 10h
111InCl3
pH 5.5
H
N
OH
111In3+
O-

Figure S5

Blot from figure 3B

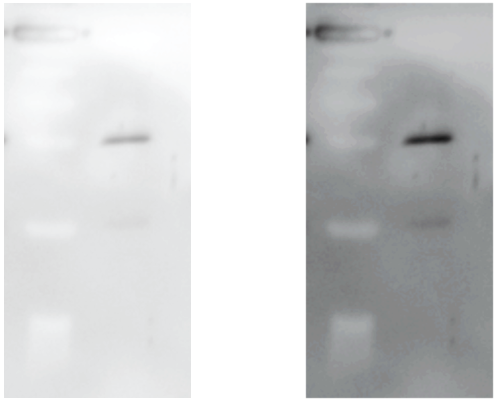

Original Modified

Pictures from figure 3c

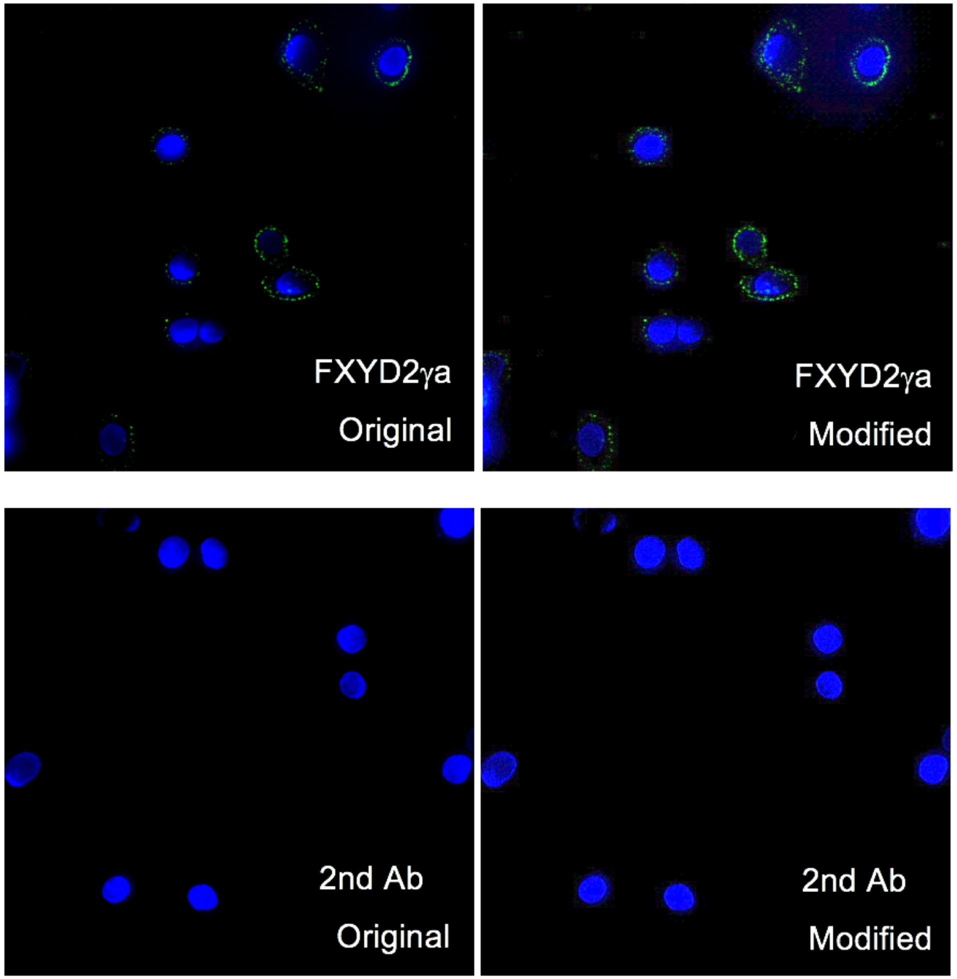

Supplementary Figure 1. MR imaging using Gd-DOTA-P88 and Gd-DOTA-Scramble in mice implanted with CHO-FXYD2γa$^+$ or wildtype CHO cells.

(A-B) Representative MR images of mice implanted with CHO-FXYD2γa$^+$ or wildtype CHO cells. Pre-contrast images were acquired before the injection of CAs and the post-contrast images were obtained about 95 minutes after i.v. administration of 0.1 mmol Gd/kg b.w. of Gd-DOTA-P88 (A) or Gd-DOTA-Scramble (B). Mice were implanted with CHO-FXYD2γa$^+$ in the right hind leg and wildtype CHO cells in the left hind leg (tumors are pointed by arrows). The images are representative of 3-4 similar experiments. Representative ROIs are drawn for xenograft (red) and noise (yellow), used for SNR quantification.

Supplementary Figure 2. Confirmation of FXYD2γa expression in CHO cells transfected with the plasmid encoding FXYD2γa.

(A) Human FXYD2γa mRNA expression was determined by qPCR in wildtype and FXYD2γa$^+$ CHO cells; n = 3, mean ± SEM. (B) A representative immunofluorescence image of FXYD2γa$^+$ and wt CHO cells stained with SPY393. The images are representative of 3 similar experiments.

Supplementary Figure 3. Non-invasive MR imaging of EndoC-βH1 tumors in mice using Gd-DOTA-P88.

Representative images of the vehicle transplantation ring in the left hind leg before (A) and 49 minutes after the i.v. administration of Gd-DOTA-P88 (B). The images are representative of 4-5 similar experiments.

Supplementary Figure 4. Coupling of P88 peptide to NOTA and radiolabeling.

Scheme for the synthesis of Gd-NOTA-P88, produced by coupling P88 to NOTA. The radiolabeling step is also depicted.

Supplementary Figure 5. Original image for Figures 3B and 3C.
